# Extensive evaluation of ATAC-seq protocols for native or formaldehyde-fixed nuclei

**DOI:** 10.1186/s12864-021-08266-x

**Published:** 2022-03-17

**Authors:** Hao Zhang, Michael E. Rice, Joseph W. Alvin, Dominique Farrera-Gaffney, James J. Galligan, Michael D. L. Johnson, Darren A. Cusanovich

**Affiliations:** 1 Department of Cellular and Molecular Medicine, University of Arizona, Tucson, AZ USA; 2 Asthma and Airway Disease Research Center, University of Arizona, Tucson, AZ USA; 3 Department of Immunobiology, University of Arizona, Tucson, AZ USA; 4 Department of Pharmacology and Toxicology, University of Arizona, Tucson, AZ USA; 5 BIO5 Institute, University of Arizona, Tucson, AZ USA; 6 Valley Fever Center for Excellence, University of Arizona, Tucson, AZ USA

**Keywords:** ATAC-seq, Chromatin accessibility, Quality control, Transcription factor, Tn5

## Abstract

**Background:**

The “Assay for Transposase Accessible Chromatin sequencing” (ATAC-seq) is an efficient and easy to implement protocol to measure chromatin accessibility that has been widely used in multiple applications studying gene regulation. While several modifications or variants of the protocol have been published since it was first described, there has not yet been an extensive evaluation of the effects of specific protocol choices head-to-head in a consistent experimental setting. In this study, we tested multiple protocol options for major ATAC-seq components (including three reaction buffers, two reaction temperatures, two enzyme sources, and the use of either native or fixed nuclei) in a well-characterized cell line. With all possible combinations of components, we created 24 experimental conditions with four replicates for each (a total of 96 samples). In addition, we tested the 12 native conditions in a primary sample type (mouse lung tissue) with two different input amounts. Through these extensive comparisons, we were able to observe the effect of different ATAC-seq conditions on data quality and to examine the utility and potential redundancy of various quality metrics.

**Results:**

In general, native samples yielded more peaks (particularly at loci not overlapping transcription start sites) than fixed samples, and the temperature at which the enzymatic reaction was carried out had a major impact on data quality metrics for both fixed and native nuclei. However, the effect of various conditions tested was not always consistent between the native and fixed samples. For example, the Nextera and Omni buffers were largely interchangeable across all other conditions, while the THS buffer resulted in markedly different profiles in native samples. In-house and commercial enzymes performed similarly.

**Conclusions:**

We found that the relationship between commonly used measures of library quality differed across temperature and fixation, and so evaluating multiple metrics in assessing the quality of a sample is recommended. Notably, we also found that these choices can bias the functional class of elements profiled and so we recommend evaluating several formulations in any new experiments. Finally, we hope the ATAC-seq workflow formulated in this study on crosslinked samples will help to profile archival clinical specimens.

**Supplementary Information:**

The online version contains supplementary material available at 10.1186/s12864-021-08266-x.

## Background

In the nuclei of eukaryotes, a fine balance has to be struck between compressing the genome to fit within strict size constraints and allowing functional elements to interact with transcription factors and effect the varied transcriptional profiles of distinct cell types present in the body [1, 2]. The end result of this balance is that most of the genome is tightly wound into nucleosomes, while some regions are left accessible to interact with proteins. Importantly, the specific regions of DNA that are made accessible change across cell types. Several assays have been developed to measure this accessibility genome-wide, including deoxyribonuclease I sequencing (DNase-seq, [3]), micrococcal nuclease sequencing (MNase-seq, [4]), and more recently the “Assay for Transposase Accessible Chromatin sequencing” (ATAC-seq, [5]). Due to lower input requirements and a simpler protocol, ATAC-seq in particular has become a popular choice to profile chromatin accessibility in a variety of settings (e.g. [6, 7]).

In ATAC-seq, nuclei are isolated and permeabilized with a detergent cocktail and then incubated with a hyperactive mutant of the Tn5 transposase to insert short oligos into the accessible regions of their chromatin and thereby simultaneously fragment DNA and “tag” the fragment ends with sequencing adapters (a process called “tagmentation”, [5]). Next, the tagmented DNA is purified, amplified, and then analyzed using next-generation sequencing. In generating and analyzing ATAC-seq data, there are a host of metrics that have been employed to evaluate data quality. During library generation, a critical step is the visualization of the amplified library either on a gel or capillary electrophoresis system (e.g. Agilent Bioanlayzer) to examine the fragment size distribution. Because Tn5 does not insert adapters into DNA wrapped around histones, ATAC-seq libraries will exhibit a characteristic nucleosomal periodicity in the fragment sizes representing successive integer numbers of nucleosomes. Too much smearing or a weak “ladder pattern” on the gel can be indicative of the disruption of chromatin integrity prior to tagmentation. Another quality control (QC) check prior to sequencing is the number of PCR cycles required to amplify the library. To avoid overamplification, the PCR reaction is usually monitored to determine the appropriate number of cycles on a real-time PCR instrument. A given sample source will generally have a consistent number of required cycles (assuming a set number of nuclei is used), and deviations from this cycle can be indicative of problems.

After sequencing, multiple data-based QC metrics have also been used to measure ATAC-seq library quality. Two commonly used metrics for quantifying signal-to-noise ratio are transcriptional start site (TSS) enrichment and fraction of reads in peaks (FRiP). The former parameter is calculated as the fold-enrichment of the peak of Tn5 insertion events near the TSS of genes genome-wide relative to the average read depth at some specified distance from the TSSs - typically 1000 or 2000 base pairs (bp) [8, 9]. The latter parameter is calculated as the fraction of all unique mapped reads that overlap some reference set of expected peaks of accessibility (this reference can be independently determined or can be identified based on the sample undergoing QC). Other metrics include a sub-nucleosomal score, which quantifies the enrichment of fragments smaller than 150 bp relative to fragments larger than 150 bp, since short fragments tend to exhibit a higher FRiP. Additionally, the percent of reads mapping to the mitochondrial genome (%mito) can be quite high depending on the protocol used. Therefore, %mito is often included as a way to measure the amount of data dedicated to chromatin accessibility versus mitochondrial coverage. Finally, the estimated library complexity is sometimes used as a proxy for enzyme efficiency.

Since 2013, when the original ATAC-seq assay was developed [5], several improvements (e.g. Omni-ATAC [10]) and variations (e.g. THS-seq [11]) have been published, including several versions with single-cell resolution [12–15]. Across this work, major components of the assay have been changed, including the lysis buffer used to isolate nuclei, the buffer used during enzymatic tagmentation of chromatin, the enzyme used (in addition to commercial enzyme, several methods for production of in-house enzyme have been published [16, 17]), and the temperature of the enzymatic reaction. There have even been modifications to work with formaldehyde-fixed samples (e.g. [18–21]). For example, ATAC-see [18] presents a protocol for fixed samples, though it was published before the Omni-ATAC modifications were implemented. In spite of all these variations, to date there has been no systematic evaluation of the effect of these different choices on the quality of the subsequent data. To fill those gaps, we evaluated the above ATAC components on both native and fixed nuclei head-to-head in a consistent experimental setting. This allowed us to directly observe the effects of individual choices and interactions between components. In addition, we explored the value of various quality control metrics in determining overall sample quality.

## Results

**Fig. 1:**
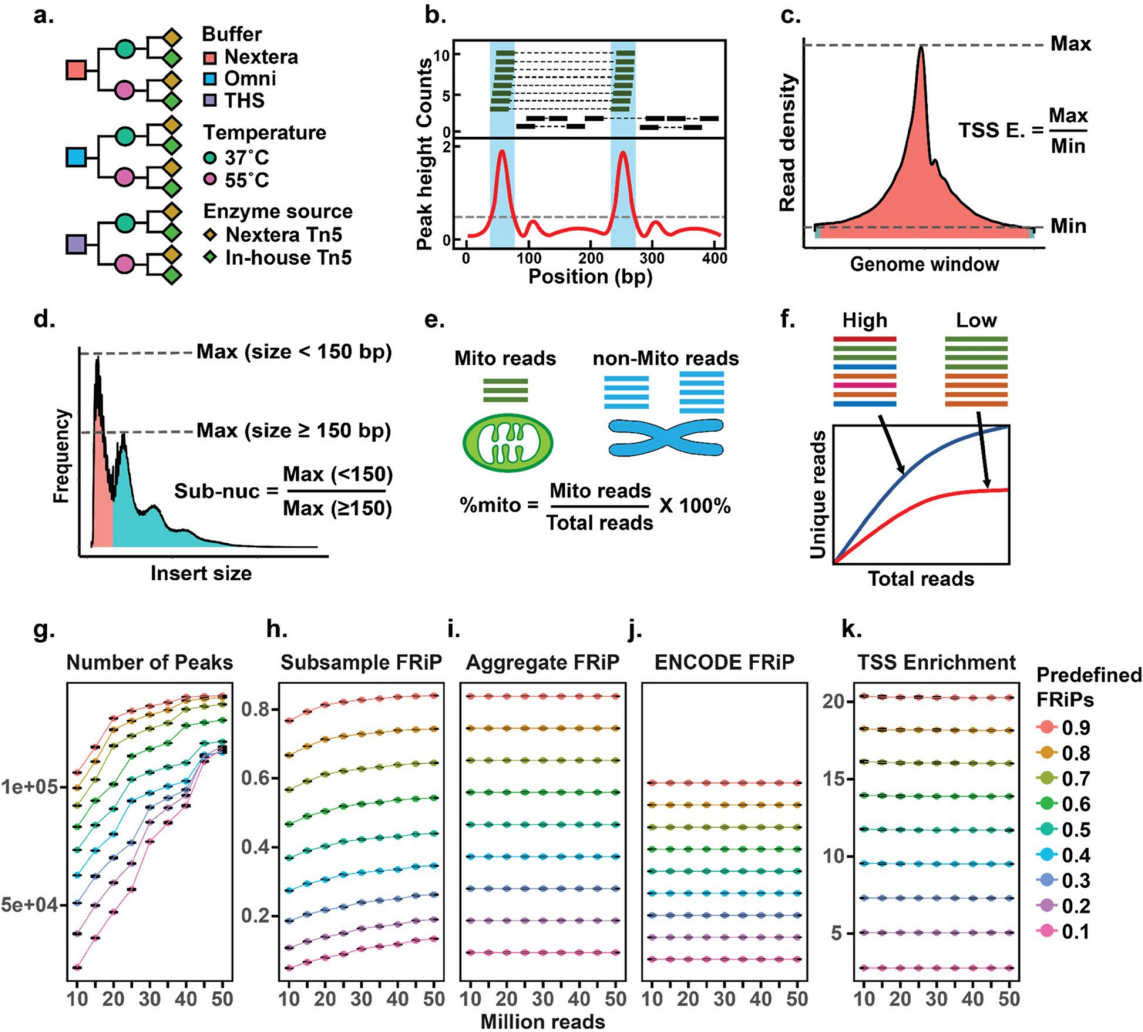
Study design schematics and evaluation of QC metrics in simulations. **a** Schematic showing all combinations of conditions tested, which included two Tn5 sources, three buffers, two temperatures all in both formaldehyde-fixed and native nuclei. **b**-**f** Schematics of main QC metrics, which included **b** FRiP, **c** TSS enrichment, **d** sub-nucleosomal score, **e** %mito, and **f** complexity (based on image from [22]). **g**-**k** Results of simulation study evaluating robustness of major QC measures. X-axes indicate the number of reads subsampled from a dataset that combined the two replicates of the GM12878 cell line from the original Omni-ATAC publication. Units of the y-axes are indicated above the plot. **h**-**j** share the same y-axis scale and units. For each subsample at a predetermined FRiP, we calculated the **g** number of peaks identified in the sample, the **h** subsample FRiP calculated on the sample, the **i** aggregate FRiP calculated using a peak set identified after aggregating all data, the **j** FRiP determined using the GM12878 DHS from ENCODE, and the **k** TSS enrichment. The dot indicates the mean of three replicate subsamples and the error bars reflect the min-max values at each simulated condition

In order to systematically compare the effect on data quality for many of the possible ATAC protocol choices in both native and crosslinked nuclei, we performed an extensive set of ATAC-seq reactions varying several components while using a single human immortalized B cell line (GM12878). Parameters tested included three different tagmentation buffers - comparing the buffer described in the Omni-ATAC protocol ([10], referred to as “Omni”), the buffer described in the THS-seq protocol ([11], referred to as “THS”), and the buffer that is supplied with commercially available Tn5 from Illumina as a part of the Nextera kit (referred to as “Nextera”). All buffers were supplemented with the same reagents as stated in the Omni-ATAC recipe (i.e. PBS, digitonin, and NP-40). In addition, we tested the temperature of the enzymatic reaction (comparing 55 °C, which is recommended for Nextera reactions, with 37 °C, which has been adopted for most ATAC-seq protocols), the source of the enzyme (comparing the commercial enzyme with the enzyme produced in-house using a modification of the protocol in [16]), and the preparation of nuclei (comparing native with formaldehyde-fixed samples). In total, we sequenced 96 samples, testing 24 different combinations of assay conditions in quadruplicate (Fig. 1a). The experiments were conducted simultaneously and all samples were sequenced to comparable depth (13 million - 27 million reads per sample, Fig. S1a) on a single run of an Illumina NextSeq and were processed through the same analysis pipeline (Methods).

### FRiP is highly dependent on the choice of peak set

**Fig. 2:**
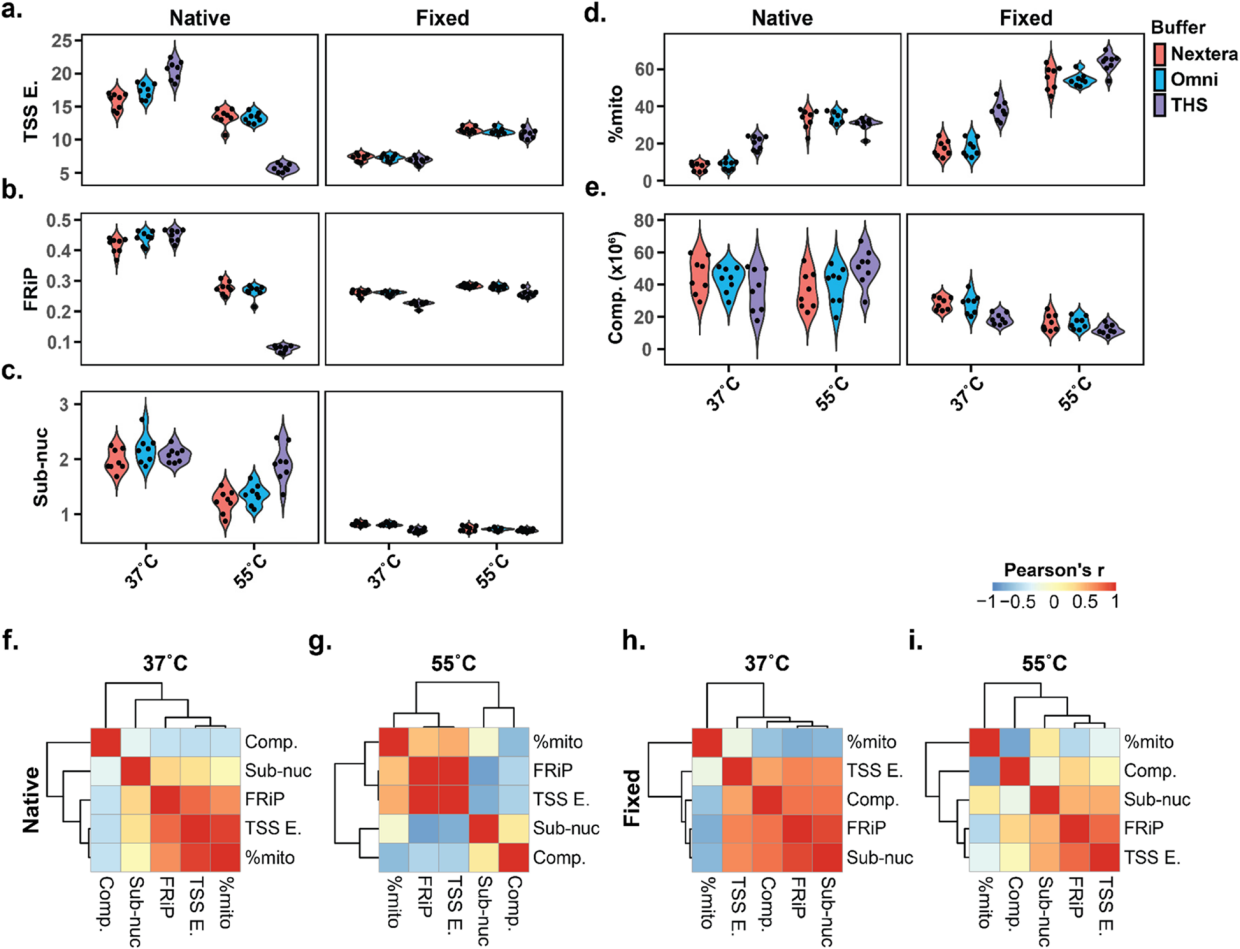
QC measures calculated for all conditions. **a**-**e** Individual metrics calculated for all 96 samples. These include **a** TSS enrichment, **b** FRiP calculated using ENCODE DNase I hypersensitive sites as the reference peaks, **c** Sub-nucleosomal score, **d** %mito, and **e** complexity. **f**-**i** Heatmaps of correlations among all 5 QC measures for **f** native samples at 37 °C, **g** native samples at 55 °C, **h** fixed samples at 37 °C, and **i** fixed samples at 55 °C

In evaluating the quality of ATAC-seq libraries, several metrics are often reported in the literature. Five commonly used measures include: FRiP (Fig. 1b), TSS enrichment (Fig. 1c, Fig. S1b, c), sub-nucleosomal score (Fig. 1d, Fig. S1d, e), %mito (Fig. 1e), and library complexity (Fig. 1f, see Methods for the calculation procedures for each QC metric). While most of the quality metrics considered are inherently sample-specific (e.g. %mito) or are defined on invariant references (e.g. TSS enrichment), FRiP can be greatly affected by how peaks are defined, and there are examples of many different ways to do so in the literature. In many studies, the peaks used in calculating FRiP are determined directly from the data generated from that study itself (e.g. [10]). However, it may not be possible or practical to generate the high sequencing depth data required for well-defined peaks. To circumvent this issue, an independently determined set of peaks is employed in other studies as a “gold standard” (e.g. using pre-existing data from a DNase-seq study to evaluate ATAC-seq data [12]). While this approach is more conservative, an appropriate independent reference is not always available. Therefore, we decided to evaluate the effect of read depth on the measure of FRiP using different references relative to TSS enrichment and sub-nucleosomal score in a contrived situation in which we simulated specific signal-to-noise ratios. To do so, we downloaded a GM12878 ATAC-seq dataset (two replicates) from the original Omni-ATAC study [10] and divided the deduplicated reads (see Methods) into separate files depending on whether or not the read pair overlapped a peak set identified from the aggregated replicates. We then subsampled read pairs from these two files to create synthetic datasets with defined FRiPs (from 10 to 90%, in 10% increments) and read depths (from 10 million to 50 million mapped unique reads (i.e. 5 million to 25 million read pairs) in 5 million read increments), and repeated this downsampling strategy three times to generate three replicate datasets at each defined FRiP and read depth. For each of those synthetic datasets we first identified peaks from the subsampled data (Fig. 1g), and then calculated the FRiP using three different sets of reference peaks: 1) peaks identified in the same subsample (“subsample FRiP”, Fig. 1h); 2) the aggregate peak set called from the original data (“aggregate FRiP”, Fig. 1i); and 3) DNase I hypersensitive sites (DHS) defined by the ENCODE consortium on the same cell line [23] (“ENCODE FRiP”, Fig. 1j). In addition, the TSS enrichment (Fig. 1k) and sub-nucleosomal score (Fig. S2a) were also quantified for each subsample (see Methods for more details on the simulation strategy). Unsurprisingly, the aggregate FRiP, ENCODE FRiP, TSS enrichment, and sub-nucleosomal score are robust to read depth and precisely controlled by the defined FRiP of the simulated library (because reads are subsampled uniformly from the library). While the ENCODE FRiP was lower than aggregate FRiP across all simulations, it was consistently so (ENCODE FRiP is ~ 0.69 times the value obtained for the aggregate FRiP, Fig. S2b), suggesting that at sufficient sequencing depth aggregate and orthogonal peak references can be considered interchangeable. In contrast, the subsample FRiP is highly correlated with the library size (Fig. 1h), because the power to detect peaks is so dependent on read depth (Fig. 1g). Furthermore, the rate at which estimated FRiP scores decay at lower sequencing depth is not consistent for various inherent library FRiPs. As a result of these analyses, we used the DNase I hypersensitive sites to calculate the FRiP for the purposes of the ATAC-seq condition comparisons described below.

### Fixation and temperature have major effects on data quality

After generating libraries on all 24 conditions (in quadruplicate), we calculated the five major quality metrics on each of our samples (Fig. 2a-e, Table S1). Although we calculated these metrics with all available reads, subsampling to 1 M reads for each sample did not meaningfully affect the QC measures (data not shown), consistent with our simulations. In general, fixed samples had lower TSS enrichments (median 14.6 for native, median 9.0 for fixed), lower FRiPs (median 0.34 for native, median 0.26 for fixed), lower sub-nucleosomal scores (median 1.9 for native, median 0.7 for fixed), higher %mito (median 24% for native, median 46% for fixed), and lower complexities (median 44.1 million for native, median 19.3 million for fixed) than native samples. We note that our %mito even with Omni buffer at 37 °C in native GM12878 nuclei is somewhat higher than the rate reported in the original Omni-ATAC protocol (median 9.5% in our experiment vs 2.0% in [10]). The temperature of the reactions had a noticeable effect on quality metrics as well (although the direction of effect was not always consistent between native and fixed samples). In native samples, the higher temperature decreased the TSS enrichments (median 17.5 for 37 °C, median 13.0 for 55 °C, Fig. 2a left panel, Fig. S1b) and FRiPs (median 0.44 for 37 °C, median 0.26 for 55 °C, Fig. 2b left panel). In contrast, for fixed samples, the higher temperature increased TSS enrichments (median 7.2 for 37 °C, median 11.2 for 55 °C, Fig. 2a right panel, Fig. S1c) while not strongly affecting the FRiP at all (median 0.26 for 37 °C, median 0.28 for 55 °C, Fig. 2b right panel). Considering the choice of buffer, Omni buffer and Nextera buffer generally performed similarly, while THS buffer had distinct consequences on quality metrics. This was particularly true in native samples at 55 °C, where THS buffer resulted in significantly lower FRiPs (*p* = 1.7 × 10^− 10^ for THS vs Nextera, and *p* = 1.1 × 10^− 9^ for THS vs Omni) and TSS enrichments (*p* = 3.3 × 10^− 8^ for THS vs Nextera, and *p* = 6.2 × 10^− 12^ for THS vs Omni) than the other two buffers. In general, the choice of enzyme had a minimal impact on quality metrics relative to the other components (Fig. S3). When testing each of the metrics across all native or fixed samples, the only significant association with enzyme source was a slight increase in complexity for native nuclei with in-house enzyme relative to commercial enzyme (fold-change = 1.24, *p* = 0.0071).

Having generated data on so many conditions in a controlled experiment, we took the opportunity to evaluate correlations among all five QC parameters pairwise across the samples. While we could observe metrics that were correlated, the same correlations were not maintained between both native and fixed samples (Fig. S4), and the observed correlation structure changed when we considered just samples processed at the same temperature (Fig. 2f-i). For example, TSS enrichment and FRiP are the two most correlated factors at 55 °C in both native and fixed nuclei (*r* = 0.99 for native at 55 °C, *r* = 0.80 for fixed cells at 55 °C, Fig. 2g, i), while TSS enrichment is most correlated with %mito at 37 °C in native nuclei (*r* = 0.93, Fig. 2f) and FRiP is most correlated with sub-nucleosomal score in fixed nuclei at 37 °C (*r* = 0.92, Fig. 2h). Interestingly, regardless of temperature, %mito was significantly negatively correlated with FRiP (*r* = − 0.73 and *p* = 5.6 × 10^− 5^ at 37 °C, *r* = − 0.51 and *p* = 0.012 at 55 °C) and estimated complexity (*r* = − 0.62 and *p* = 1.1 × 10^− 3^ at 37 °C, *r* = − 0.77 and *p* = 1.0 × 10^− 5^ at 55 °C) in fixed samples, but was significantly positively correlated with FRiP (*r* = 0.65 and *p* = 5.3 × 10^− 4^ at 37 °C, *r* = 0.44 and *p* = 0.031 at 55 °C) while remaining significantly negatively correlated with complexity in native samples (*r* = − 0.45 and *p* = 0.026 at 37 °C, *r* = − 0.66 and *p* = 4.1 × 10^− 4^ at 55 °C). However, we interpret the apparent correlation between FRiP and %mito as an artifact driven by the fact that THS buffer-containing reactions provide substantially different QC metrics from the other conditions.

It is worth noting that all of these analyses were conducted after removing duplicate read pairs. In some cases, removing duplicate reads may be technically difficult or undesired. It is possible that not removing duplicate reads could affect QC metrics. To evaluate this, we re-analyzed our data without first removing duplicate reads (Fig. S5). In our case, the QC metrics were largely unaffected, except for some subtle changes for sub-nucleosomal score in fixed nuclei at 55 °C (Fig. S5c right panel). However, we note that there were relatively few PCR duplicates at our sequencing depth, and so this observation may not hold for all data sets.

### Effects of buffers and temperatures are relatively consistent in other sample types

Because these experiments were conducted in a single cell line, we wanted to evaluate if the relationships among QC metrics would be consistent across sample types. To test this, we repeated the experimental conditions using native nuclei isolated from lung tissue from three different mice (Fig. S6a-e, k, Table S1). We found that the patterns of FRiP and TSS enrichment scores across all conditions were highly consistent between cell line and primary cells, with subtle differences in sub-nucleosomal score (Fig. S6c) and a major reduction in %mito (Fig. S6d), suggesting FRiP and TSS enrichment scores in particular are reliable QC metrics for various cell type sources.

In many experimental settings, researchers may have limited input material. To address whether the effects we observed were robust with respect to the number of input nuclei, we also repeated the lung experiment (triplicates representing 12 native conditions) using 1000 nuclei as input instead of the 20,000 we used in all other experiments (Fig. S6f-j, l, Table S1). Again, the trends were consistent, although the variance between replicates was greatly increased.

### The ability to identify accessible peaks is greatly influenced by experimental variables

One of the major tasks of ATAC-seq data analysis is the identification of peaks of accessibility. These peaks demarcate the regulatory elements important for encoding cellular function. Identifying those elements that change in response to various stimuli can inform us of how the genome regulates cellular responses. Given the extensive differences in QC metrics, we wondered how that would impact the ability to identify peaks in individual conditions. To consider this, we combined all 4 replicates for each condition tested in GM12878, subsampled all combined libraries down to the minimum number of deduplicated reads observed for any condition (~ 6 million reads per condition), and then identified peaks using MACS2 [24] (Table S2; see Methods for more details).

For native nuclei, we identified a median of 56,529 peaks per sample across conditions, with Nextera buffer at 37 °C and commercial enzyme uncovering the most peaks (70,665) and THS buffer at 55 °C and in-house enzyme uncovering the least (19,618). For fixed nuclei, we observed a median of 50,684 peaks across all conditions, and the same two conditions at the extremes - Nextera buffer at 37 °C and commercial enzyme uncovering 54,504 peaks and THS buffer at 55 °C and in-house enzyme uncovering 46,632, although the standard deviation across conditions was much lower for fixed nuclei than native nuclei (2330 vs 18,257, for fixed and native respectively). While the sequencing depth of these samples was lower, they appeared to match well with the number of peaks identified in our simulations (when subsampling 10 million reads), given their FRiP scores. In fact, a correlation with a Pearson’s r of 0.98 was observed in native samples between the number of peaks and the FRiP based on the DHS peak set for native nuclei (Fig. S7a). The FRiP and peak number in fixed samples were also correlated after stratifying by temperature (*r* = 0.96 for 37 °C and 0.98 for 55 °C, Fig. S7b), but no correlation was observed when considering all conditions.

To confirm that peak calling after removing duplicate reads was appropriate, we re-ran the peak calling pipeline without first removing duplicates. We found that in almost all conditions (except for THS buffer at 55 °C in native cells, which had very low numbers of peaks overall) the vast majority of peaks were identified in both settings with only a few peaks gained or lost (Fig. S7c, d). While it is common to treat the bulk ATAC data as a quantitative feature (generating a matrix of read counts overlapping individual peaks for all samples, akin to common RNA-seq analysis pipelines), some consider ATAC data as a binary vector (presence or absence of peaks). To address these disparate ways of looking at the data, we evaluated the peak calls using first a qualitative framework and then a quantitative framework.

Given the large differences in the number of peaks identified in each condition, we sought to explore if there were any biological factors that might help to explain the loss of power to identify peaks in one condition relative to another. In order to do so, we first examined the reproducibility of peaks identified in each condition, which we defined as the presence of a detectable peak in at least three different conditions. We set the threshold for reproducibility at three because the performance of the two enzymes (Nextera and in-house) was so similar that they tend to have highly overlapping peak sets across conditions. In both native and fixed nuclei, a large majority of peaks were reproducible across all conditions, except that THS buffer at 55 °C identified fewer peaks in native samples and a large proportion of peaks in this condition were not reproducible in other conditions (Fig. S7e, f). We further downloaded all ChIP-seq peak sets available from ENCODE for transcription factors (TFs) profiled in unstimulated GM12878 and used a hypergeometric testing framework to identify enrichments of overlap with ENCODE ChIP-seq peaks for ATAC-seq peaks that were “missing” (i.e. that were reproducibly identified in three other conditions, but not observed in the condition of interest; see Methods for details). In total, 73 different ChIP-seq peak sets were available and designated as “good” quality by ENCODE (Table S3). This analysis revealed a subtle (yet significant) enrichment for overlap with ﻿CCCTC-Binding Factor (CTCF) and cohesin components across most conditions at 37 °C (Nextera and Omni buffers at 37 °C for native samples and all 37 °C conditions for fixed samples; Fig. S8a, b; Table S4). Read density plots of these regions indicate that the “missed” sites are generally lowly accessible across all conditions (Fig. S8c, d). We interpret these results as indicating that in general peaks not detectable in any given condition are not biased towards a specific class of regulatory element. However, there appears to be a subtle reduction in power to detect a small subset of CTCF and cohesin sites at 37 °C.

A common quantitative framework for analysis of transcriptomic and epigenomic data is to consider the correlation between replicate samples in the quantitative measures of a set of commonly defined features (such as genes or a peak set). To enable this, the peaks identified in each condition were merged to establish a common peak set that was used to generate a matrix of the number of reads overlapping each peak for each sample. This matrix of quantitative peak accessibility was transformed with a variance stabilizing transformation algorithm in DESeq2 (see Methods), and then we conducted principal component analysis (PCA) and hierarchical clustering (visualized with a heatmap) to evaluate how the samples correlated with each other (see Methods). We did this for the fixed and native samples separately. Analyzing the data this way indicated the same structure as observed by the individual quality metrics. Overall, the correlation among samples was high (Pearson’s r ranges: 0.88–0.98 for native samples and 0.91–0.96 for fixed samples), however the samples clustered based first on reaction temperature and then by THS buffer versus the other two buffers for both native and fixed cells (Fig. S9a, b). For PCA, the first component stratified the native samples by both temperature and THS and accounted for 85% of the observed variation (Fig. S9c). For fixed samples, the first component captured 31% of the variation and stratified samples by reaction temperature as well, while THS was separated from other conditions along the second principal component (Fig. S9d).

### Reaction temperature and buffer bias regulatory element detection

**Fig. 3:**
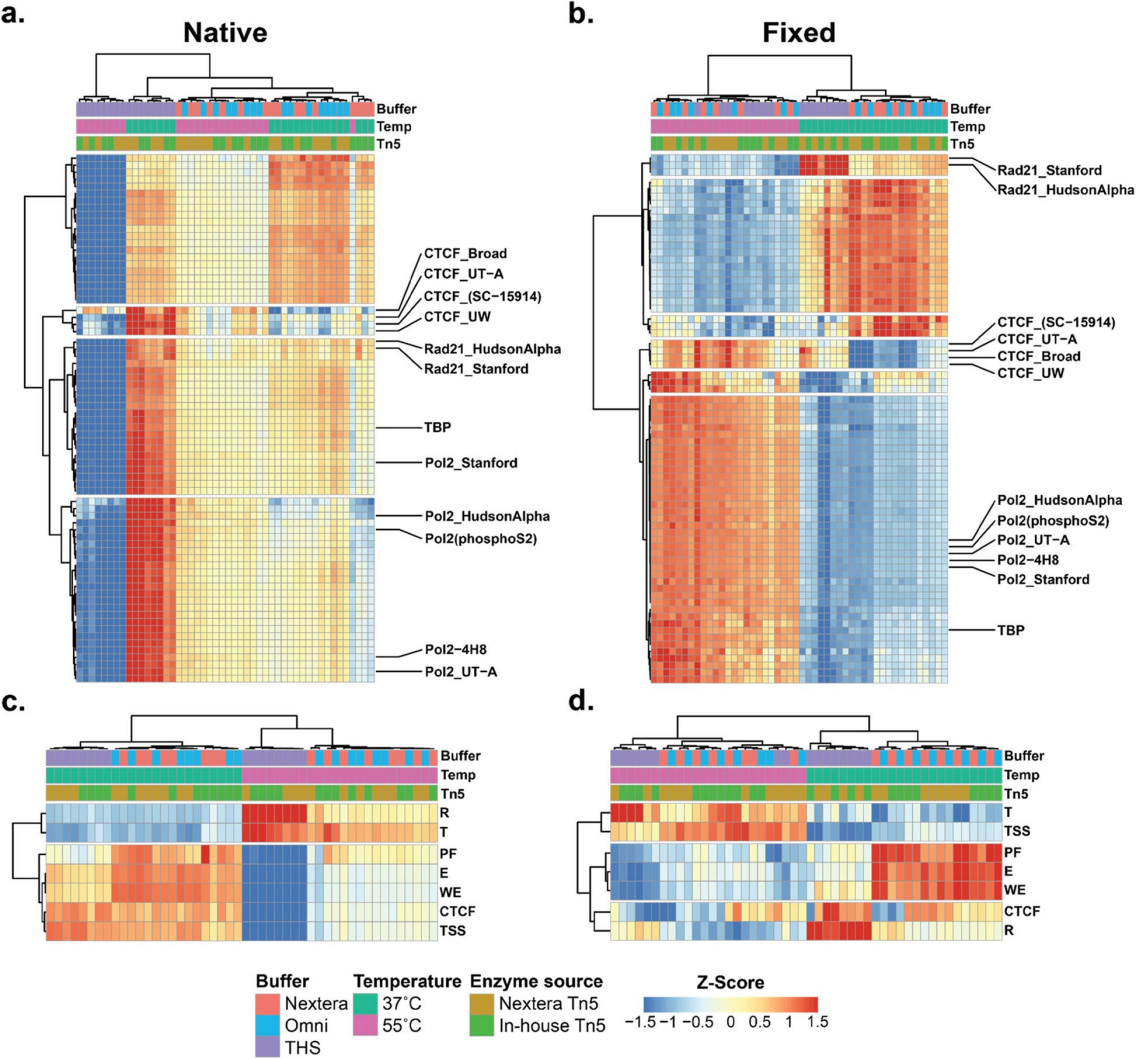
Coverage of TFs and genome segments varies by condition. **a** Heatmap of row-normalized coverage (native samples) for 73 ChIP-seq datasets from ENCODE. Z-scores capped at +/− 1.5. Color bars above heatmaps indicate conditions. **b** Heatmap of row-normalized coverage (fixed samples). **c** Heatmap of row-normalized coverage for ENCODE genome segments (native samples). **d** Heatmap of row-normalized coverage for ENCODE genome segments (fixed samples). Abbreviations in (**c**, **d**) - TSS: Predicted promoter region including TSS; E: Predicted enhancer; WE: Predicted weak enhancer or open chromatin cis regulatory element; CTCF: CTCF enriched element; R: Predicted Repressed/Low Activity region; T: Predicted transcribed region; PF: Predicted promoter flanking region

Given the results above and noting that peaks from different classes of regulatory elements are not equally accessible [25], we wondered if these differences in the ability to identify peaks might impact the functional annotations that might be enriched in peak sets from various conditions. To explore this further, we returned to the ChIP-seq peak sets we had downloaded from ENCODE (Table S3), and evaluated how well our ATAC-seq data covered these TF peaks. We first examined how well each of our ATAC-seq samples overlapped with ChIP-seq data in general. To do so, we merged the peaks from all ChIP data and intersected our ATAC-seq reads with those merged peaks - calculating the Fraction of Reads in Peaks from all ChIP data (FRiP-ChIP, Fig. S10a, b). We found that the pattern of variation for FRiP-ChIP across all samples strongly resembled the pattern of FRiP scores, which is attributable to the high degree of overlap between ChIP-seq peaks and DHS peaks. To distinguish between signal compression and potential bias in functional regions covered by ATAC-seq in the various protocols, we next intersected our ATAC-seq data with individual TF peak sets. In this case we divided the number of reads overlapping ChIP peaks for a particular TF by the number of reads overlapping any ChIP-seq peak to calculate the Fraction of Reads for a specific TF (FRiP-TF). We chose this approach because our simulations indicated that intersecting sample reads with external peak calls is robust to read depth. After normalizing the FRiP-TF scores row-wise (i.e. by transcription factor), we hierarchically clustered the samples to identify trends in the coverage of different TFs across the various conditions (see Tables S5 and S6 for the matrix of FRiP-TF score in native and fixed samples, respectively). For native samples, we found that the major axis of variation involved an interaction between THS buffer and temperature (Fig. 3a): THS buffer samples at 55 °C were an outlier for poor coverage across all TF peak sets, while THS buffer samples at 37 °C had the highest coverage for most TF peak sets (relative to other buffers at 37 °C), including a cluster of CTCF ChIP-seq peak sets (cluster 4, Table S5) and two clusters of factors that included components of the cohesin complex and general TFs such as TATA-binding protein (TBP) and Pol II (clusters 2 and 3, Table S5). The other two buffers (Omni and Nextera) were largely indistinguishable from each other and showed the best enrichment for a cluster of enhancer-associated factors (including p300) at 37 °C (cluster 1, Table S5). For fixed nuclei, the picture was different to some extent (Fig. 3b). In this context, samples clustered first by temperature. As with the native nuclei, THS buffer resulted in different profiles than the other two buffers (although the effect was less apparent at 55 °C). Tagmentation at 37 °C provided better coverage for enhancer-associated features (cluster 1, Table S6), while tagmentation at 55 °C increased coverage at CTCF sites and promoter-associated factors (clusters 2 and 4, Table S6). One difference for fixed nuclei was that cohesin-associated regions were better covered at 37 °C (cluster 6, Table S6). Considering genome segmentation tracks produced by ENCODE presented a slightly different picture: in native samples, tagmentation at 37 °C did a better job covering all classes of segmentation, except for repressive elements and regions of transcription. In fixed samples, 37 °C better covered all classes of ENCODE genome segmentations except for TSSs and regions of transcription (Fig. 3c, d).

Because the standardized values can distort the actual variation observed across samples, we calculated the median absolute deviation (MAD), a robust measure of variability, for each factor. Overall, the amount of variation in FRiP-TF was ≤1% for most samples for any given factor (median MAD = 0.007 for native samples, median MAD = 0.010 for fixed samples; Fig. S10c), suggesting that changes in coverage of particular factors in different conditions are subtle even though they are pervasive. In comparison to the FRiP-TF, the native samples showed higher variance across different segments (median MAD = 0.020), while the fixed samples did not (median MAD = 0.005, Fig. S10d).

### chromVAR identifies enriched motifs in specific conditions

**Fig. 4:**
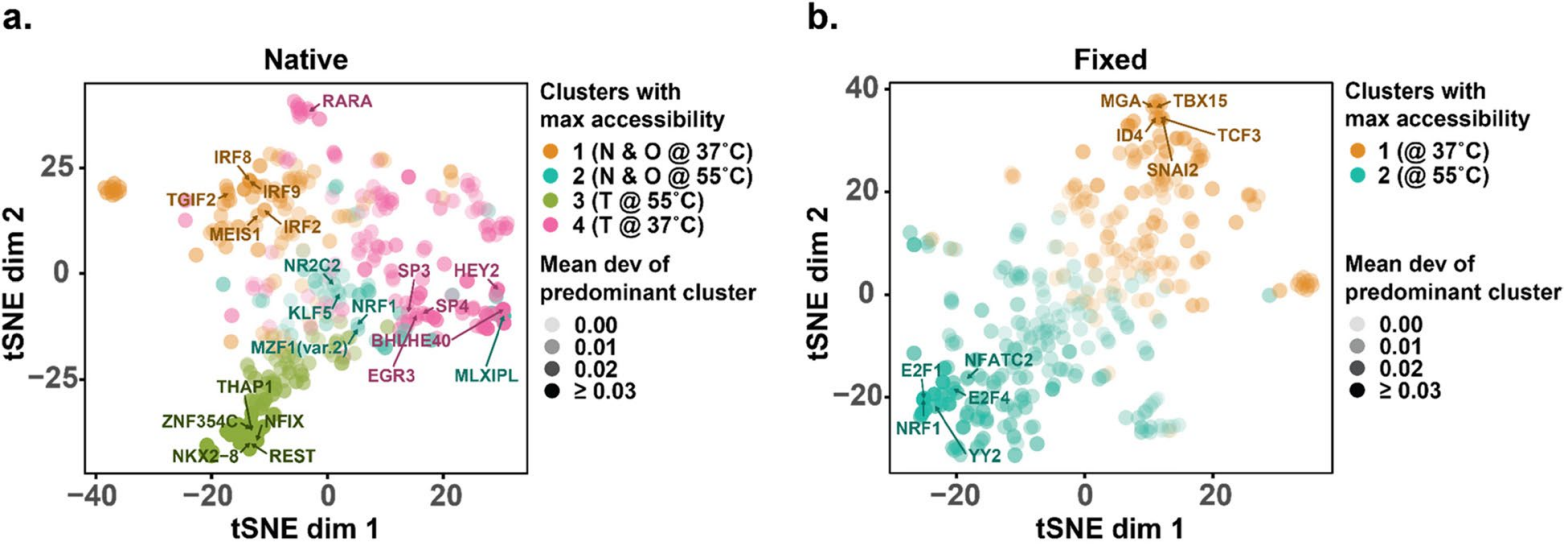
t-SNE of motif usage across conditions for both native and fixed samples. Each point represents an individual motif. After generating deviation scores for all 386 motifs across all conditions, t-SNE was used to visualize the relationship between motifs. Points are colored by the cluster of conditions (defined by hierarchical clustering of samples) in which that motif is most enriched. The top five most enriched motifs for each cluster are annotated on the plot. For native samples (**a**), four clusters of conditions were identified: “cluster 1” = Nextera and Omni buffers at 37 °C (orange), “cluster 2” = Nextera and Omni buffers at 55 °C (blue), “cluster 3” = THS buffer at 55 °C (green), “cluster 4” = THS buffer at 37 °C (pink). For fixed samples (**b**), two clusters of conditions were identified: “cluster 1” = 37 °C (orange), “cluster 2” = 55 °C (blue). The mean deviation score of the cluster each motif was most enriched in is indicated by the level of transparency. Abbreviations: N: Nextera buffer; O: Omni buffer; T: THS buffer

Because these effects on ChIP-seq peak and genome segmentation coverage were somewhat unexpected, we decided to take a theoretically less biased, orthogonal approach to validate the observation. To do so, we implemented the chromVAR pipeline [26] to score coverage of individual motifs genome-wide and looked at whether specific motifs had distinct patterns of accessibility that correlated with experimental conditions. chromVAR transforms ATAC-seq data by annotating each peak of accessibility with the presence or absence of a database of TF binding motifs and then calculating the deviation from expected coverage for individual motifs genome-wide. These deviations are also corrected for GC content bias and sparsity through simulations. In this way, chromVAR uses a normalized, background-corrected matrix of the number of reads overlapping peaks that contain specific motifs summarized across the genome as input. The framework is designed to work with single-cell chromatin accessibility data and therefore we reasoned that it should be robust for bulk data with ~ 1 to 7 million reads per sample, as in our case. In principle, this approach should correct for artifacts in motif enrichment that are driven by inherent differences in FRiP and read depth in the different conditions. Clustering samples on chromVAR deviation scores assorted native samples into four clusters representative of the Omni and Nextera buffers at the two temperatures and distinct clusters for THS buffer at the two temperatures (Fig. S11a). Fixed samples were sorted into two main clusters indicative of temperature alone (Fig. S11b). To visualize the similarity of usage patterns for individual motifs across the samples, we generated a t-stochastic neighbor embedding (t-SNE) of the deviation scores in chromVAR. This analysis indicated that individual motifs exhibited differential patterns of accessibility across conditions and that groups of motifs were most enriched in specific conditions (Fig. 4). Stratifying by the major clusters of conditions identified in this analysis (four for native samples and two for fixed samples), we tested for significant differences in deviation scores for each motif and found that many motifs had significant changes in coverage for specific clusters at an adjusted *p*-value threshold of 1 × 10^− 3^. In native samples (Fig. S12a-d and Table S7), the cluster representing Omni and Nextera at 37 °C (“cluster 1”, Fig. S12a) had 80 motifs with significantly increased accessibility and 61 motifs with significantly decreased accessibility relative to the other samples. The cluster representing Omni and Nextera at 55 °C (“cluster 2”, Fig. S12b) had 59 motifs with increased accessibility and 12 motifs with decreased accessibility at the designated threshold. The cluster representing THS 55 °C (“cluster 3”, Fig. S12c) had 42 motifs with significant increased accessibility (notably, the transcriptional repressor REST was ranked in the top 5), and 163 motifs with significant decreased accessibility. The cluster representing THS at 37 °C (“cluster 4”, Fig. S12d) had 80 motifs with increased accessibility and 21 motifs with decreased accessibility. In fixed nuclei, which only had two clusters, we identified 80 motifs with significantly increased accessibility at 37 °C and 124 motifs with significantly increased accessibility at 55 °C (Fig. S12e and Table S8).

## Discussion

Since 2013, there have been many different variations in the ATAC-seq protocol reported, including THS-seq and Omni-ATAC. In addition, groups have reported protocols for working with both native and fixed nuclei, and even different reaction temperatures (both 37 °C and 55 °C). Furthermore, there are a variety of ways to evaluate the quality of the data generated by ATAC-seq. To date, there has not been a systematic evaluation of many of these variables, or of the QC measures employed. To facilitate a better understanding of how protocol choices impact QC metrics and the utility of the quality metrics themselves, we designed a series of experiments using a common cell line and a matrix of conditions: three reaction buffers (Omni, Nextera, and THS), two reaction temperatures (37 °C and 55 °C), two enzyme sources (in-house and commercial), and two fixation states (native and fixed). In addition, we tested the 12 native conditions in nuclei isolated from mouse lung tissue (also considering two different nuclei input numbers in this case).

We determined that Omni and Nextera buffers performed best across two different sample types, were largely interchangeable with each other (regardless of the other conditions), and that those buffers with reactions at 37 °C for native nuclei consistently provide the highest data quality. While we have not evaluated individual components here, one possible explanation for the differences observed in THS buffer at 55 °C in native nuclei is the presence of potassium in the buffer, which has previously been shown to affect chromatin compaction [27]. For fixed samples, 55 °C seemed to outperform 37 °C, except that the fraction of the library dedicated to mitochondrial reads was significantly higher at 55 °C. We were somewhat surprised by this observation, as some of our previous single-cell work (in Drosophila embryos, [20]) with fixed samples tagmented at 55 °C had very low mtDNA yields. The difference in species notwithstanding, this raises the possibility that washing the nuclei after tagmentation (prior to reverse crosslinking) may eliminate some of the contaminating mtDNA. It is also worth noting however that this higher mitochondrial burden may be desirable in some instances, given the recent demonstrations that mtDNA mutations can be used to trace cell lineage and infer pathogenic cell states [28–30]. In general, native samples allowed for the identification of more peaks than fixed samples, even at the same read depth. Finally, the differences between commercial enzyme and in-house enzyme were minor.

We also took the opportunity to evaluate how related individual QC metrics were, including: TSS enrichment, FRiP, sub-nucleosomal score, % mitochondrial reads, and estimated complexity. While there were correlations between them in certain conditions, the relationships were complicated and there are apparent interactions between the tested conditions that affect how these metrics are related. Fixation, temperature, and the use of THS buffer had the largest apparent effects on the QC metric relationships. Taken together, we recommend several metrics for evaluating any new samples, particularly TSS enrichment (which does not require an experimentally determined reference), FRiP (which is most directly related to peak calling), and %mito (which indicates the fraction of data generated that is informative), as these appeared the most informative in our experience. Sub-nucleosomal score in particular seems to be an inconsistent indicator of data quality. We suspected it might be useful for several reasons: (1) it was correlated with FRiP and TSS enrichment in our simulations (Fig. S2a), (2) the original ATAC-seq paper noted an enrichment of TSS-overlapping reads from sub-nucleosomal fragments [5], and (3) a common single-cell ATAC-seq analysis pipeline uses a related metric to filter out low-quality cells [31]. While we found sub-nucleosomal score correlated with FRiP and TSS enrichment within a condition, it was unreliable in our data across conditions and cell types (e.g. Fig. 2 and Fig. S6). Therefore, we do not recommend its use as a QC metric.

Finally, we noted that the choice of buffer and temperature had profound effects on the biology observed - skewing how well our data covered peaks of different functional classes and individual TF motifs. This discrepancy was not explained simply by power to detect individual peaks, as one of the methods we employed (chromVAR) was explicitly designed to control for this kind of confounding. Instead, it seems that the chromatin accessibility itself is affected by experimental choices, which influences the ability of Tn5 to insert. In light of this, researchers should take care in deciding on the appropriate protocol for their system and especially in comparing results from different studies.

There are several caveats to this work. The first is that we only evaluated conditions in one cell line and one tissue. It is likely that different cell types will be influenced by different components, and so researchers should consider trying several protocols when working with a new cell type. Second, because we tested so many conditions, we did not sequence each library as deeply as would be typical for biological experiments. We strove to design the downstream analysis to be robust to read depth, but it is possible that this influences some of our results.

## Conclusions

In summary, we have identified a set of preferred conditions for performing ATAC-seq (Omni or Nextera buffer at 37 °C in native nuclei) and recommend a set of QC metrics (rather than any individual one) for evaluating the data (including TSS enrichment, FRiP, and %mito). In addition, we provide a detailed protocol for working with formaldehyde-fixed samples updated for use with the Omni-ATAC modifications that we hope will be useful to the community. Finally, we have uncovered artifacts that may lead to subtle batch effects that should be taken into account when designing ATAC-seq experiments.

## Methods

### Cell culture conditions and fixation

The GM12878 cell line (Coriell Cell Repository) was cultured at 37 °C with 5% CO_2_ in RPMI 1640 medium (GIBCO, cat. no. 11875–093) containing 15% FBS (GIBCO, cat. no. 10437–028), 100 U/ml Penicillin Streptomycin (GIBCO, cat. no. 15140–122). Cells were counted and split to 300,000 cells/ml three times a week, and ~ 2 million cells were harvested on Day 4 after waking culture to perform ATAC-seq and all cells were harvested 1 week later for fixation. Each replicate of ATAC-seq data was generated with independent cell stocks, but the native and fixed datasets shared the same cell stocks.

On the day of fixation, cells were washed and resuspended in 9.2 ml of RPMI medium without additives, then crosslinked with 270 μl of 37% formaldehyde (1% final, VWR, cat. no. MK501602) for 10 min at room temperature. The fixation reaction was quenched by adding 500 μl of 2.5 M glycine (0.125 M final, Sigma, 50,046-50G) and incubated for 5 min at room temperature, and then on ice for 15 min. The fixed cells were collected and resuspended in the freezing buffer, which contains 50 mM Tris-HCI (pH 8.0, Invitrogen, cat. no. 15568025), 5 mM Magnesium Acetate (Sigma, cat. no. 63052), 25% glycerol (VWR, cat. no. RC3290–32), 0.1 mM EDTA (Fisher, cat. no. AM9260G), 5 mM DTT (Fisher, cat. no. P2325), 2% protease inhibitor (Sigma, cat. no. P8340). Then, 2 million cells per aliquot were flash frozen in liquid nitrogen for long-term storage.

### Tn5 production and purification

To produce the in-house Tn5, we adapted the strategy of using an N-terminal 6xHis-Sumo3 tag based on the protocol in [16]. First, we reconstructed an expression plasmid containing the sequences described in the original paper. We did so by using the pTXB1-Tn5 plasmid (Addgene #60240) as a template to amplify the Tn5 DNA with the primer sequences of 5′-GATCGGATCCATGATTACCAGTGCACTGCATCG-3′ and 5′-GATCAAGCTTTTAGATTTTAATGCCCTGCGCC-3′. The PCR product was digested with BamHI and HindIII, and then ligated into a pETM11-SUMO-SNCA-GFP plasmid (Addgene #107292) that had been linearized by the BamHI and HindIII digestion as well. This plasmid now containing Tn5 in-frame with the 6xHis-Sumo3 tag was then transferred to the BL21 (DE3) codon + RIL *E. coli* strain (Stratagene) for protein expression. The protein expression and purification process was a modification of protocol described in [16]. First, a single colony was picked from an agar plate to inoculate a starter culture in 50 ml of Luria Broth (LB) growth media with 30 μg/ml kanamycin and 34 μg/ml chloramphenicol. The starter was cultured at 37 °C overnight with shaking at 225 rpm. On the following day we monitored the growth of the culture by measuring optical density at 600 nm (OD_600_). When the OD_600_ of the starter culture reached 0.5, we diluted the culture in 1 L of Terrific Broth (TB) supplemented with 1% glucose and 0.4% glycerol to an OD_600_ of 0.05 in a 2.8 L flask. We then monitored the culture for ~ 2 h until it reached an OD_600_ of 0.5. At this point, the culture was transferred to 18 °C (shaking at 225 rpm) for 30 min. After equilibration, IPTG (final concentration of 0.2 mM) was added to the larger culture to induce expression of Tn5. After culturing overnight at 18 °C, cells were harvested by centrifuging at 14,000 r.c.f for 30 min at 4 °C, decanting the media, and frozen as a pellet at − 80 °C. Cell pellets were resuspended in running buffer (50 mM Tris pH 7.4, 800 mM NaCl, 20 mM imidazole, 1 mM TCEP and 10% glycerol) supplemented with protease inhibitors (final concentrations: 20 μM Leupeptin, 14 μM Pepstatin A, 2 mM PMSF), lysed by sonication (VirTis Virsonic 600) in the presence of 10 μg/ml Bovine Pancreatic DNase, cleared by centrifuging 30,000 r.c.f for 30 min at 4 °C, and the cell lysate was concentrated using an Amicon Ultra-15 Centrifugal Filter Unit (10,000 NMWL). The sample was then loaded on an equilibrated His-Tag purification column (HisTrap FF Crude 5 ml, Cytiva) on a Akta Pure chromatography system to collect Tn5 protein. After washing the column with running buffer, Tn5 was eluted off the column with elution buffer (50 mM Tris pH 7.4, 800 mM NaCl, 250 mM imidazole, 1 mM TCEP and 10% glycerol). Fractions were analyzed via SDS-PAGE and the fractions containing Tn5 were pooled. To remove the 6xHis-Sumo3 tag, we added the 6xHis-tagged SenP2 to the pooled elution fractions in a 1:1000 mass ratio and incubated overnight at 4 °C. The digested enzyme was loaded onto another His-Tag purification column to collect the untagged Tn5 as flow-through and remove SenP2. The digested Tn5 was then concentrated and equilibrated into a buffer containing 20 mM MES (2-(N-Morpholino)ethanesulfonic acid) pH 6 and 100 mM NaCl, and then further purified using cationic exchange (UNOsphere S-column) followed by size exclusion chromatography (Superdex® 200 Increase 10/300 GL, Sigma-Aldrich, cat. no. GE28–9909-44) equilibrated with SEC buffer (50 mM Tris pH 7.5, 800 mM NaCl, 10% glycerol, 0.2 mM EDTA, 2 mM DTT). Finally, the enzyme purity was checked via SDS-PAGE. Purified enzyme was diluted to 0.6 mg/ml in SEC buffer and then diluted 1:1 with 100% glycerol supplemented with 800 mM NaCl, for a final storage buffer concentration of 25 mM Tris pH 7.5, 800 mM NaCl, 0.1 mM EDTA, 1 mM DTT and 55% glycerol. The final product was stored at − 20 °C.

### Tn5 loading

The linker oligos Tn5ME-A, Tn5ME-B and Tn5MErev (Table 1) from [17] were ordered from IDT and resuspended in the annealing buffer containing 50 mM NaCl, 40 mM Tris-HCl pH 8.0 at a final concentration of 100 μM. The procedure of annealing linker oligos and loading them to Tn5 followed the protocol as described in [16]. Briefly, one volume of Tn5ME-A or Tn5ME-B was mixed with one volume of Tn5MErev, and then incubated with the following PCR program: 95 °C for 5 min; slowly cool down to 65 °C (0.1 °C/sec); 65 °C for 5 min; then slowly cool down to 4 °C (0.1 °C/sec). The annealed linker oligos were stored at − 20 °C. To load the Tn5, we added 1 μl of each annealed linker (50 μM) to 20 μl of the Tn5 stock (0.3 mg/ml), and incubated the oligo-Tn5 mixture at 23 °C for 30 min with shaking in an Eppendorf thermomixer at 350 rpm. The loaded Tn5 was stored at − 20 °C for no longer than 2 months before the experiment.

**Table 1:** The sequences of Tn5 linker oligos

Linker oligo	Sequence 5′ - > 3′
Tn5ME-A	TCGTCGGCAGCGTCAGATGTGTATAAGAGACAG
Tn5ME-B	GTCTCGTGGGCTCGGAGATGTGTATAAGAGACAG
Tn5MErev	[phos]CTGTCTCTTATACACATCT

### ATAC-seq on native GM12878 nuclei

We adopted the general Omni-ATAC protocol [10] but modified the components according to our designs (Fig. 1A). All centrifuging steps were performed at 500 r.c.f for 5 min at 4 °C in a fixed-angle centrifuge, unless specified. In general, ~ 2 million native cells were collected and washed using 4 ml 1X PBS (pH 7.4, Gibco, cat. no. 10–010-023) supplemented with 0.04% BSA (PBSB), and then resuspended in 200 μl of ATAC-seq lysis buffer. Lysis buffer was made by supplementing ATAC resuspension buffer (RSB) with detergents (see below). RSB buffer is 10 mM Tris-HCl (pH 7.5, Invitrogen, cat. no. 15567027), 10 mM NaCl (Invitrogen, cat. no. AM9759) and 3 mM MgCl_2_ (Invitrogen, cat. no. AM9530G) in nuclease free water. RSB was made in bulk and stored at 4 °C long-term. On the day of the experiment, the ATAC lysis buffer was made by adding 0.1% IGEPAL (Sigma, cat. no. I3021), 0.01% digitonin (Invitrogen, cat. no. BN2006), and 0.1% Tween-20 (Bio-Rad, cat. no. 1610781) to RSB. Detergent percentages reported are final concentrations. After resuspending cell pellets in the lysis buffer, they were incubated on ice for 3 min, and then the lysis was stopped by adding 1 ml RSB containing 0.1% Tween-20. Nuclei were then centrifuged at 500 r.c.f for 10 min at 4 °C and resuspended in 200 ul of PBSB. The nuclei were counted and diluted to 3100 nuclei/μl with PBSB. Since the nuclei are sensitive to osmotic pressure and will inflate in Trypan blue solution, we found that adding nuclei to 2X Omni buffer (see Table 2) followed by adding 1 volume of Trypan blue improves the integrity of the nuclei for counting. 6.6 μl of diluted nuclei (20,460 total nuclei) were transferred to 12.4 μl of transposition reaction (10 μl 2X buffer (see Table 2 for detailed recipe for each buffer), 0.2 μl of 1% digitonin, 0.2 μl of 10% Tween-20, and 2 μl of H_2_O). We then added 1 μl of transposase to the transposition mix containing nuclei. Note that we had previously determined the relative efficiency of the two enzymes via qPCR (Fig. S13). Based on this titration, the in-house Tn5 was first diluted to 87 μg/ml with water and then 1 μl was added to the tagmentation reaction. 1 μl of the commercial enzyme was added as is. Tagmentation was carried out on a thermocycler at either 37 °C or 55 °C for 30 min. Tagmented DNA was cleaned up using Zymo DNA Clean and Concentrator-5 columns with 5X binding buffer (Zymo, cat. no. D4004). 5 μl of purified DNA out of 10 μl purified product was amplified in a 25 μl PCR reaction containing NEBNext PCR master mix (1X final), 0.5X SYBR Green and 1.25 μM of Ad1 primer from [5] and 1.25 μM of an N7 primer containing custom barcodes (Table S9). Samples were amplified on a Bio-Rad CFX Connect Real-time cycler using the following program: 72 °C for 5 min; 98 °C for 30s; cycling at 98 °C for 10 s, 63 °C for 30 s, 72 °C for 1 min; samples were monitored and stopped when it appeared that their exponential amplification was leveling off. At this point the samples were allowed to incubate at 72 °C for an additional minute to fully extend the library. All native samples were stopped after 9 cycles and fixed samples were stopped after 10 cycles. PCR products were cleaned up with AMPure XP beads. A double size selection was performed to remove DNA fragments larger than ~ 1500 bp (with 0.4X AMPure XP beads) and smaller than ~ 100 bp (with 1.5X AMPure XP beads). To do this, we added 25 μl of Qiagen Buffer EB to each PCR reaction to bring the volume to 50 μl, and then added 20 μl of beads (homogenizing the mixture well by pipetting), followed by a 5 min incubation at RT. The samples were then put on a magnet stand to bind the beads. 68 μl of the supernatant was transferred to a new 1.5 ml tube and an additional 53 μl of beads were added to the supernatant and resuspended thoroughly (we estimated that 19.4 μl of the 68 μl transferred supernatant was bead buffer and 48.6 μl was sample, so to get to 1.5X beads for the second selection we needed to add an additional 72.9–19.4 μl of beads, which we rounded off to 53 μl). After another 5 min at RT, the samples were placed on the magnet to clear the beads and the supernatant was discarded. Beads were washed twice with 200 μl of 80% EtOH (made fresh for each experiment). After the second wash, the beads were briefly spun and any residual ethanol was removed. The beads were then put back on the magnet stand and air dried for 1 min. Then, beads were removed from the magnet and resuspended in 22 μl of Buffer EB, incubated for 2 min at RT and then placed on the magnet stand again. 20 μl of supernatant was transferred to a new tube. Following this AMPure size selection, the ATAC-seq libraries were quantified by Qubit 1X dsDNA HS Assay Kit and run on a 6% PAGE gel prior to sequencing.

**Table 2:** Recipes for buffers tested

Buffer	Recipe
2X Nextera	Purchased from Illumina (cat. no. 20034197)
2X Omni	20 mM Tris HCl (pH 7.5), 10 mM MgCl_2_ and 20% Dimethyl Formamide [10]
2X THS	66 mM Tris acetate (pH 7.8), 132 mM Potassium acetate, 20 mM Magnesium acetate, and 32% Dimethyl Formamide [11]

Buffer column provides the name used, and the Recipe column specifies final concentrations for a 2X buffer. For Omni and THS, the original source of the recipe is cited

### ATAC-seq on fixed GM12878 nuclei

The frozen, fixed cells (~ 2 million) were incubated in a 37 °C water bath until thawed (~ 1 min). After thawing, the cells stored in 1 ml freezing buffer were diluted with 3 ml of PBSB. The fixed cells were collected by centrifuging at 500 r.c.f for 10 min at 4 °C, resuspended again in 1 ml PBSB, and then centrifuged at 500 r.c.f for 5 min at 4 °C to wash the cells. Following washing, the nuclei were isolated and tagmented as described in the ATAC-seq protocol for native samples described above. After tagmentation, crosslinks were then reversed for the fixed, tagmented nuclei by adding 60 μl of reverse-crosslinking buffer containing 0.067% SDS and 1.33 mg/ml Proteinase K (Qiagen, cat. no. 19133) in Qiagen Buffer EB (final concentration of 0.05% SDS and 1 mg/ml Proteinase K) directly to the tagmentation reactions, and incubating the samples at 65 °C for 15 h in a thermomixer with shaking at 1000 r.p.m. Following crosslink reversal, tagmented DNA was purified, amplified and size-selected as described in the ATAC-seq protocol for native samples.

### Mouse lung dissection and storage

Mice were euthanized via exsanguination confirmed by cervical dislocation. All animal activity was approved by the University of Arizona IACUC. Whole mouse lungs were dissected from 3 male C57BL/6 J mice that were 24 weeks old. The dissected tissues were flash frozen in liquid nitrogen and then transferred to − 80 °C for long-term storage.

### Nuclei isolation from mouse lungs

The nuclei isolation procedure was performed following the single-nucleus isolation protocol described in [32]. In brief, we cut a ~ 0.1–0.2 g piece from the lung sample removed from − 80 °C and kept it on dry ice until use. The tissue block was thawed almost completely on ice for 1 min, and then injected with 1 ml of cell lysis buffer, which was made of 1x cOmplete protease inhibitor cocktail (1 tablet per 10 ml solution, Sigma-Aldrich, Cat. 11,836,153,001) in Nuclei EZ prep buffer (Sigma-Aldrich, Cat. NUC101), into the center of the tissue with a 30G needle and syringe. Following lysis buffer injection, the tissue was chopped into small pieces with scissors and then transferred along with the lysing buffer into a gentleMACS C tube (Miltenyi Biotec, Cat. 130–096-334). An additional 1 ml of lysing buffer was added into the C tube to make a final volume of 2 ml. The minced tissue was then homogenized using a gentleMACS tissue dissociator by running the ‘m_lung_01’ program followed by the first 20 s of the ‘m_lung_02’ program. After homogenization, tissue lysate was briefly centrifuged to reduce foam and then passed through a 40 μm cell strainer in a 50 ml tube. After passing the sample through, the strainer was rinsed with 4 ml of washing buffer (PBS with 1% BSA). The nuclei were counted with Trypan blue in the presence of 2X Omni buffer (see “ATAC-seq on native GM12878 nuclei” for details), and centrifuged at 500 r.c.f for 5 min at 4 °C. Then, we removed the supernatant and resuspended the nuclei to a concentration of 4–5 million nuclei/ml in a nuclei freezing buffer containing 50 mM Tris-HCI (pH 8.0, Invitrogen, cat. no. 15568025), 5 mM Magnesium Acetate (Sigma, cat. no. 63052), 25% glycerol (VWR, cat. no. RC3290–32), 0.1 mM EDTA (Fisher, cat. no. AM9260G), 5 mM DTT (Fisher, cat. no. P2325), and 2% protease inhibitor (Sigma, cat. no. P8340). 1 ml aliquots of the nuclei were flash frozen in liquid nitrogen and then transferred to a liquid nitrogen dewar for long-term storage.

### ATAC-seq on mouse lung nuclei

The mouse lung nuclei were removed from the liquid nitrogen dewar and thawed in a water bath at 37 °C for 1 to 2 min until only a tiny ice crystal remained. Then, we transferred the 1 ml of nuclei stored in freezing buffer to a 15 ml tube containing 3 ml RSB washing buffer (RSB supplemented with 0.1% Tween-20 and 0.1% BSA), centrifuged nuclei at 500 r.c.f for 10 min in a pre-chilled (4 °C) swinging-bucket centrifuge, and then removed supernatant. After supernatant removal, the nuclei were resuspended with 1 ml RSB washing buffer and then filtered with a 40 μm Flowmi Cell Strainer (SP BEL-ART, cat. no. 136800040) followed by centrifugation at 500 r.c.f for 5 min at 4 °C. Again, the supernatant was aspirated, and the nuclei were resuspended in 150 ul PBSB and then counted with Trypan blue as mentioned before. After counting, the nuclei were diluted either to a concentration of 20,000 nuclei per 6.6 ul or 1000 nuclei per 6.6 ul with PBSB, and then processed with 12 ATAC-seq protocols (3 tagmentation buffers by 2 transposition temperatures by 2 Tn5 enzymes) as described in the section describing “ATAC-seq on native GM12878 nuclei”, generating a total of 24 ATAC libraries for each replicate mouse lung. The sample barcodes used for each condition and replicate are listed in Table S9.

### Sequencing

The 96 ATAC-seq libraries of the GM12878 cell line and 72 libraries of mouse lungs were pooled separately and sequenced with 2 × 76 bp reads in two independent runs on an NextSeq 550 Platform using the High Output Kits.

### ATAC-seq data analysis

The specific programs (and their version) used in data analysis were as follows: Trimmomatic v0.36 [33], SAMtools v1.4 [34], Picard v2.20.2 [35], Bowtie2 v2.2.9 [35, 36], MACS2 v2.1.2 [24], bedtools v2.28.0 [24, 37], deepTools v3.5.1 [38], R v4.0.4 [39], DESeq2 [40], and chromVAR [26].

The paired-end reads were preprocessed using trimmomatic to trim the Nextera adaptors and low-quality reads with the parameter setting as “LEADING:3 TRAILING:3 SLIDINGWINDOW:4:10 MINLEN:20”. The trimmed reads were then mapped to either the hg19 human genome or mm10 mouse genome reference contingent upon the sample source using Bowtie2. The parameters “-X 2000” and “-3 1” were used to restrict the maximum fragment length of 2000 bp and trim 1 base from the 3′ end of each read before alignment (we found a perfect match between forward and reverse reads from a read-pair could lead to inaccurate library quality metrics). Following mapping, only the reads confidently (MAPQ ≥10) mapped to assembled nuclear chromosomes, and in proper pairs (we used the “-f3” and “-F12” options in SAMtools) were preserved for downstream analysis. Picard “MarkDuplicates” was then used to remove duplicate reads and estimate library complexity.

### Peak calling

To call peaks with deduplicated reads, we combined nuclear genome-mapped reads for all 4 replicates from the same condition and subsampled each combined file to the minimum number of reads observed for any condition (6,046,132). Then, MACS2 was used to call peaks with deduplicated bed files, considering a 200 bp window centered on the read start using the parameters ﻿"--nomodel --keep-dup all --extsize 200 --shift -100". Because each peak may have multiple summits (and will therefore be listed multiple times in the resulting peak bed file), the peaks output from MACS2 were then merged into a single peak set for each sample using bedtools “merge”. To examine the effects of deduplication on peak calling, we subsampled 6,254,654 reads (including duplicates) from each sample, which is the minimum number of reads observed for any condition, and then called peaks using MACS2 with the "--keep-dup auto" option. The peaks shared by two peak calling protocols and the peaks unique to each were identified by bedtools "merge" with the "collapse" function.

### Calculation of ATAC-seq QC metrics

#### FRiP

The FRiP score was determined using a merged peak set that combined two replicates of GM12878 DHS hotspots obtained from the ENCODE consortium (ENCFF235KUD and ENCFF491BOT) [23]. The reads overlapping the DHS peak reference were counted using bedtools “intersect” with “-u” option. Mitochondrial reads were removed before calculating FRiP.

#### TSS enrichment

The human and mouse TSS coordinates were obtained from the Gencode human reference v38 [41] and Gencode mouse reference vM23 [42], respectively. To build TSS references, we first collected the most upstream base (accounting for strand) of each transcript using a custom R script, and then only the TSSs of gene types and transcript types listing the following terms were included: “protein_coding”, “lncRNA”, “IG_C_gene”, “IG_D_gene”, “IG_J_gene”, “IG_LV_gene”, “IG_V_gene”, “IG_V_pseudogene”, “IG_J_pseudogene”, “IG_C_pseudogene”, “TR_C_gene”, “TR_D_gene”, “TR_J_gene”, “TR_V_gene”, “TR_V_pseudogene”, “TR_J_pseudogene”. We also excluded transcripts with a tag of “readthrough_transcript” or “PAR”. These filters were similar to the filtering strategy used by the 10X single-cell ATAC-seq pipeline [43]. The TSS enrichment score was calculated as defined by ENCODE [8]. Briefly, the read depth at each position of a 4000 bp window centered on the TSSs was calculated, and then normalized by dividing by the average read depth in the 100 bp at each end of this window in 1 bp bins. The TSS enrichment score was determined by the maximum value of the normalized read depth in the 200 bp upstream of the TSS.

#### Sub-nucleosomal score

The matrices of insert size distribution were created by Picard “CollectInsertSizeMetrics” using deduplicated reads. For the simulation results, insert sizes were actually calculated directly from the bed files. The sub-nucleosomal score is calculated by the ratio between the maximum fragment counts < 150 bp and the maximum fragment counts > 149 bp.

#### Estimated complexity

As described in ATAC-seq data analysis, the library complexity was estimated by Picard “MarkDuplicates” after filtering out unmapped and mitochondrial reads.

#### %mito

The percent mitochondrial reads was calculated by dividing the total number of reads mapping to the mitochondrial genome by the total number of reads after adaptor trimming (note that this calculation occurs before removing duplicate reads).

### Simulation of ATAC-seq QC metrics

The two replicates of GM12878 ATAC-seq data were downloaded as raw fastq files from the Omni-ATAC study ([10]; SRA accessions: SRR5427887 for GM12878-OmniATAC-RepA and SRR5427886 for GM12878-OmniATAC-RepB), and processed as described in the ATAC-seq data analysis section, except without optical duplicate detection during deduplication since the read names were not available in the raw fastq files. The deduplicated bam files from each replicate were merged using SAMtools "merge", and then converted into a single bed file to call peaks. The repetitive peaks resulting from different summits were further collapsed with bedtools “merge” to construct a master peak set. We then intersected the read pairs in the bed file with the master peak set to identify read pairs that overlapped peaks. The read pairs with at least one end overlapping the master peak set were assigned to the “signal” set and the read pairs with neither end overlapping the master peak set were assigned to the “background” set using bedtools “pairToBed”. Subsequently, we subsampled either “signal” reads or “noise” reads from these two sets and merged them to create a series of synthetic datasets with defined FRiPs (10 to 90% in 10% increments) and read depths (from 10 million to 50 million reads in 5 million read increments). Reads were kept in pairs during the subsampling process. For each of the synthetic datasets, we identified intrinsic peaks for each of them, calculated the FRiPs determined either by the master peak set (“aggregate FRiP”) or intrinsic peak set (“subsample FRiP”), and quantified TSS enrichment and sub-nucleosomal score as described in the calculation of ATAC-seq QC metrics.

### Functional enrichment analysis

#### ChIP-seq enrichment

For ChIP-seq enrichments, we downloaded all available unstimulated GM12878 ChIP-seq datasets that were designated as “good” quality by ENCODE [44]. The analysis for enrichments of overlap with ChIP-seq peaks for ATAC-seq peaks “missing” from each condition was performed with a hypergeometric test using the "phyper" function in R. The background rate of overlap was determined using a master ATAC peak set that merged all peaks called in each condition and filtered out any peaks only observed in one or two conditions. The bedtools "merge" function was used to merge peaks. Peaks that overlapped by even 1 bp were considered the same peak in this analysis. The *p*-value determined by the hypergeometric test was adjusted for multiple testing using the "BH" method [45] implemented in the "p.adjust" function in R. The adjusted p-value cutoff to determine significant enrichment was 0.05. The fold-enrichment for overlap with each ChIP-seq dataset was calculated by dividing the frequency of “missing” ATAC-seq peaks overlapped by ChIP-seq peaks in each condition by the frequency of ATAC-seq peaks overlapped by ChIP-seq peaks observed in the master peak set. Overlaps were determined by the bedtools "intersect" function.

To visualize the read density across ChIP-seq peaks, we first generated a coverage track (bigWig) for each condition that concatenated the deduplicated reads from all 4 replicates using the "bamCoverage" module from deepTools [38] with the following parameter settings: "--binSize 1; --normalizeUsing CPM". For read density plots, we standardized all ChIP-seq peak windows to a 4000 bp window centered at the midpoint of the peaks, and evaluated the read coverage across all those genomic regions using "computeMatrix" (deepTools). Regions with zero read depth across all ATAC-seq samples were excluded. The "plotHeatmap" function (deepTools) was used to visualize the read density at each region. Read density plots were sorted in descending order by the mean read density value per region.

For calculating enrichments for each sample, we calculated the number of reads overlapping with each ChIP-seq dataset using bedtools “intersect” with the “-u” option, and then divided these counts by the total number of reads that overlapped with any ChIP-seq peak set to calculate the FRiP-TF for each TF. The hierarchical clustering and heatmap visualization were generated using the "pheatmap" package in R and implementing the “ward.D2” clustering method.

#### Genome segmentation enrichment

A consensus merge of the genome segmentations produced by the ChromHMM and Segway software was obtained from ENCODE [46]. For each sample, the first base at the 5′ end of each read was intersected with the genome segmentation tracks using bedtools “intersect” to annotate the reads with one of the seven different segmentation categories. The number of reads within each category was divided by the total number of deduplicated reads for each sample.

### Principal component analysis

To create a common set of peaks for PCA, we combined all replicates from the same condition and downsampled them to 6,046,132 reads (the minimum number of unique reads observed for any condition) for each condition to call peaks with MACS2. After that, all peaks were merged together to form a non-overlapping peak set using bedtools “merge”. A peak-count matrix was then generated using deepTools “multiBamSummary” with peaks as rows and samples as columns. The count data was log_2_-transformed and normalized with respect to library size using the “rlog” function in DESeq2, and then the top 5000 most variable sites were used to conduct PCA. The log-transformed counts were also used to calculate pairwise sample correlation across all peak sites for hierarchical clustering and heatmap visualization of samples.

### ChromVAR

For chromVAR analysis, we resized all peaks identified from each condition to a uniform width of 500 bp, centered them at the summit, and removed overlapping peaks using the “readNarrowpeaks” function in chromVAR. A matrix of fragment counts per sample for each peak was then created with deduplicated reads, and used as an input for the chromVAR analysis pipeline. The human motifs were obtained from JASPAR core database and used to annotate the peaks. “Background” peak sets that comprise similar GC content and average accessibility for each peak were created and used to compute a bias-corrected deviation for each motif with the default chromVAR settings. Then, we used the bias-corrected deviation to cluster samples and identify significantly differentially accessible motifs for each cluster following the standard chromVAR pipeline. To visualize motif similarity, we performed t-SNE using the “deviationsTsne” function with “perplexity” equal to 10.

## Supplementary Information

**Additional file 1: Supplementary Table S1.** The QC metrics for both the GM12878 cell line and mouse lungs. Column 1 indicates the sample ID. Columns 2-4 show the ATAC components. Columns 5-8 specify the fixation state, nuclei input, replicate ID, and sample type. Columns 9-15 show the QC metrics.

**Additional file 2: Supplementary Table S2.**. Peaks identified in each ATAC-seq protocol in the GM12878 cell line. Column 1 lists the chromosome of each peak. Column 2 lists the start coordinate of each peak. Column 3 lists the end coordinate of each peak. Columns 4-7 list the conditions (fixation state, buffer, temperature, and enzyme) that peak was identified in.

**Additional file 3: Supplementary Table S3.** List of GM12878 ChIP-seq data. Column 1 (“TF”) indicates the name and source, if provided, of transcription factors. Column 2 lists the links for downloading each GM12878 ChIP-seq dataset.

**Additional file 4: Supplementary Table S4.** Hypergeometric tests. Column 1 (“TF”) indicates the name and source, if provided, of transcription factors. Columns 2 and 3 show the p value and adjusted p value calculated by Benjamini & Hochberg method. Column 4 indicates the fold enrichment. Column 5 shows the number of condition-specific “missing” peaks overlapping with each ChIP-seq data. Column 6 shows the number of “missing” ATAC-seq peaks in each condition. Column 7 indicates the number of background peaks overlapping with each ChIP-seq data. Column 8 shows the total number of background peaks for either native or fixed samples. Column 9 shows the number of ChIP-seq peaks. Columns 10-13 indicate the fixation state, buffer, temperature, and Tn5 enzyme, respectively. The “missing” ATAC-seq peaks were defined as the peaks that were observed in at least three other conditions, but not observed in the condition of interest. The background peaks indicate the peaks shared among at least three conditions and calculated separately for native and fixed samples. The fold enrichment for overlap with each ChIP-seq dataset was calculated by dividing the frequency of ATAC-seq peaks overlapped by ChIP-seq peaks observed in each condition (the ratio of Column 5 to Column 6) by the frequency of ATAC-seq peaks overlapped by ChIP-seq peaks observed in the background peak set (the ratio of Column 7 to Column 8).

**Additional file 5: Supplementary Table S5.** The matrix of FRiP-TF score in native samples and the clusters assigned to each ChIP-seq dataset. Column 1 (“TF”) indicates the name and source, if provided, of transcription factors. Column 2 lists the cluster membership for each TF. Columns 3-50 show the sample ID for each ATAC condition as listed in Table S1.

**Additional file 6: Supplementary Table S6.** The matrix of FRiP-TF score in fixed samples and the clusters assigned to each ChIP-seq dataset. Column 1 (“TF”) indicates the name and source, if provided, of transcription factors. Column 2 lists the cluster membership for each TF. Columns 3-50 show the sample ID for each ATAC condition as listed in Table S1.

**Additional file 7: Supplementary Table S7.** Differential motif deviation analysis results generated using chromVAR in native samples. Files contain results for differential deviation score tests between clusters for all motifs. Column 1 is the human motif ID from JASPAR CORE database; Column 2 shows the cluster of interest for each differential test; Columns 3 and 4 indicate the p value and adjusted p value calculated by the differentialDeviations function in chromVAR; Column 5 shows the difference between the mean of bias corrected deviations in the cluster of interest and the mean of bias corrected deviations in all other clusters.

**Additional file 8: Supplementary Table S8.** Differential motif deviation analysis results generated using chromVAR in fixed samples. Files contain results for differential deviation score tests between clusters for all motifs. Column 1 is the human motif ID from JASPAR CORE database; Columns 2 and 3 indicate the p value and adjusted p value calculated by the differentialDeviations function in chromVAR; Column 4 shows the difference between the mean of bias corrected deviations in 37°C samples and the mean of bias corrected deviations in 55°C samples.

**Additional file 9: Supplementary Table S9.** List of ATAC-seq primers used for PCR and corresponding samples for each barcode. The Nextera N5 primer (Ad1_noMX) and the N7 primer “Ad2.1” and other N7 primer sequences (besides the barcodes) were obtained from [5]. Column 1 (“Name”) indicates the name we used for each primer. Column 2 (“Barcode in Fastq”) shows the sequence returned by the sequencer for that particular primer. Column 3 (“Oligo”) shows the sequence of the primers. Columns 4-11 specify the sample ID, buffer, transposition temperature, enzyme source, fixation state, nuclei input, replicate ID, and sample type for each sample indexed by the corresponding barcode.

## Data Availability

ATAC-seq data generated for this study have been deposited in Gene Expression Omnibus (GSE174280).

## Abbreviations

ATAC-seq: Assay for Transposase Accessible Chromatin sequencing
FRiP: Fraction of Reads in Peaks
TSS: Transcription Start Site
TSS E: TSS enrichment
Sub-nuc: Sub-nucleosomal score
%mito: The percent of reads mapping to the mitochondrial genome
Comp: Library complexity
FRiP-ChIP: Fraction of Reads in Peaks from ChIP-seq data
FRiP-TF: Fraction of Reads in Peaks from ChIP-seq data for a specific TF
PCA: Principal component analysis
t-SNE: t-Stochastic Neighbor Embedding
